# CNN-Based Identification of Pathogens of Concern in Shrimp

**DOI:** 10.3390/ani15213194

**Published:** 2025-11-03

**Authors:** Tharyar Aung, Rapeepun Vanichviriyakit, Kittisak Chayantrakom, Somkid Amornsamankul, Pallop Huabsomboon

**Affiliations:** 1 Department of Mathematics, Faculty of Science, Mahidol University, Bangkok 10400, Thailand; tharyar.au1@student.mahidol.edu (T.A.); kittisak.cha@mahidol.ac.th (K.C.); somkid.amo@mahidol.ac.th (S.A.); 2 Department of Anatomy, Faculty of Science, Mahidol University, Bangkok 10400, Thailand; rapeepun.van@mahidol.ac.th; 3 Center of Excellence for Shrimp Molecular Biology and Biotechnology, Faculty of Science, Mahidol University, Bangkok 10400, Thailand; 4 Center of Excellence in Mathematics, CHE, Bangkok 10400, Thailand

**Keywords:** CNN, deep learning, transfer learning, EfficientNet, MobileNet, image classification, computer-aided diagnosis

## Abstract

**Simple Summary:**

Shrimp farming plays an important role in global food production, but it is often threatened by diseases that damage the shrimp’s digestive organ, the hepatopancreas. These diseases can cause slow growth, weakness, or even sudden death, leading to serious financial losses for farmers. Traditional diagnosis requires laboratory tests, which are costly, slow, and not always available in small-scale farms. In this study, we used modern computer vision, a form of artificial intelligence that learns to recognize patterns in images to help identify three major shrimp diseases from microscope slides of tissue samples. We tested two lightweight computer models, MobileNet and EfficientNet, to see how well they could recognize diseased tissue compared to healthy tissue. Both models performed very well, achieving over 95% accuracy with MobileNet proving especially fast and efficient. These results show that artificial intelligence could be used as a practical, affordable tool for farmers and veterinarians to diagnose shrimp diseases quickly, even in areas with limited laboratory facilities.

**Abstract:**

Concerning shrimp diseases, including acute hepatopancreatic necrosis disease (AHPND), hepatopancreatic parvovirus (HPV) infection and *Enterocytozoon hepatopenaei* (EHP) microsporidiosis negatively impact shrimp aquaculture through acute mortality, chronic growth retardation or compromised health that increases susceptibility to concurrent infections. All three diseases damage hepatopancreas, a vital organ for nutrient absorption and growth, though their clinical outcomes differ: AHPND is typically associated with rapid, high mortality, EHP primarily causes chronic production losses and HPV, while currently of lower pathogenic significance, may still impair health under certain conditions. Outbreak severity is often intensified by poor water quality, inadequate farm management, antibiotic misuse and pathogen vectors, leading to substantial economic losses. Timely and accurate diagnosis is therefore critical for effective disease management. This study investigates two convolutional neural network (CNN) architectures, EfficientNet and MobileNet. A curated and preprocessed dataset was used to fine-tune both models with a standardized custom classification head, ensuring a controlled backbone comparison. Experimental results show both architectures achieving over 95% accuracy, with MobileNet providing faster inference suitable for on-site deployment. These findings demonstrate the practical feasibility of lightweight CNN-based diagnostics tools for real-time, scalable, and cost-efficient health monitoring in shrimp aquaculture, bridging the gap between the laboratory-grade performance and field-level usability.

## 1. Introduction

Shrimp aquaculture is increasingly threatened by infectious diseases that target internal organs essential for shrimp health and production. Among the most concerning are acute hepatopancreatic necrosis disease (AHPND), hepatopancreatic microsporidosis (HPM) caused by *Enterocytozoon hepatopenaei* (EHP) and hepatopancreatic parvovirus (HPV) infection. These pathogens primarily affect the hepatopancreas, a vital organ responsible for digestion and nutrient absorption in shrimp.

**Figure 1:**
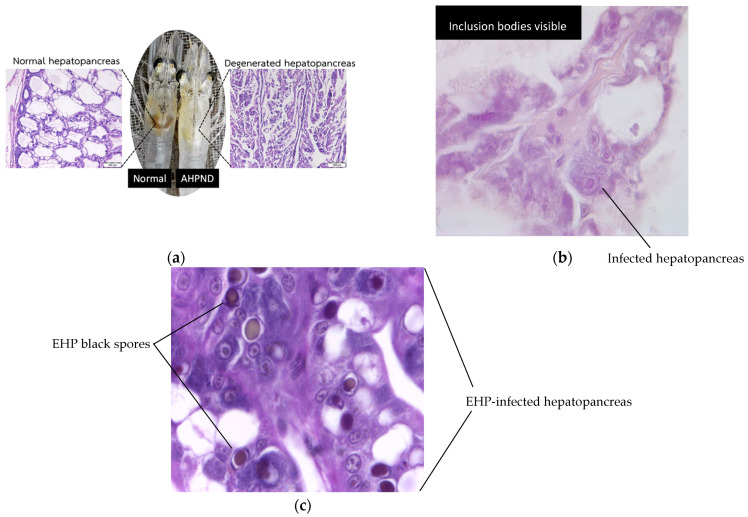
Representative histopathological images of shrimp hepatopancreas tissues. (**a**) Acute hepatopancreatic necrosis disease (AHPND): comparison between normal hepatopancreas (**left**) and degenerated hepatopancreas (**right**) from Vibrio parahaemolyticus-infected shrimp showing several epithelial cells sloughing and tubule atrophy. (**b**) Hepatopancreatic parvovirus (HPV) infection caused by Penaeus monodon densovirus (PmoDNV): hypertrophied nuclei with distinct basophilic intranuclear inclusion bodies. (**c**) Hepatopancreatic microsporidiosis (HPM) caused by *Enterocytozoon hepatopenaei* (EHP): presence of intracellular spores within hepatopancreatic epithelial cells and free spores in tubule lumens. All samples were stained with hematoxylin and eosin for microscopic examination.

AHPND, caused by Vibrio parahaemolyticus, can result in severe hepatopancreatic atrophy and massive sloughing of epithelial cells, often leading to high mortality during early cultivation stages [1,2]. Hepatopancreatic microsporidiosis (HPM) caused by *Enterocytozoon hepatopenaei* (EHP) is a chronic, production-limiting infection that reduces nutrient absorption and growth performance, though it is not typically associated with acute mass mortalities [3,4,5,6,7]. Histologically, EHP infection is characterized by intracellular spores within hepatopancreatic epithelial cells and free spores in tubule lumens. HPV caused by Penaeus monodon densovirus (PmoDNV) is detected histologically by basophilic intranuclear inclusion bodies in hypertrophied hepatopancreatic nuclei [8,9,10]. While HPV has been reported widely in Asia, its current impact on farmed shrimp health is considered low in most regions and may often be subclinical. Representative histopathological features of AHPND, EHP, and HPV infections are shown in Figure 1.

To combat these diseases, researchers have increasingly turned to automated image analysis for high-precision diagnostic support. Traditional image processing methods, while useful, often suffer from limited efficiency and accuracy due to the need for manual feature engineering. In recent years, both machine learning and deep learning approaches, especially convolutional neural networks (CNNs), have been employed to enhance classification performance [11,12,13]. CNNs offer end-to-end feature learning directly from images with minimal preprocessing, enabling early and reliable diagnosis of shrimp hepatopancreatic diseases.

Previous studies have explored a range of diagnostic and management strategies for hepatopancreatic diseases in shrimp aquaculture. In mid-2021, Kumar et al. [14] critically evaluated the use of antibiotics to manage Acute Hepatopancreatic Necrosis Disease (AHPND). They found that overuse of antibiotics can increase shrimp susceptibility to secondary viral infections and accelerate antimicrobial resistance. Their work emphasized economic risks and proposed integrated alternatives including probiotics, phage therapy, plant-based bio-actives and habitat management to reduce disease incidence.

Later in 2021, Dhar and Mai [7] conducted a comprehensive review of bacterial and viral pathogens implicated in severe hepatopancreatic diseases, particularly AHPND and hepatopancreatic microsporidiosis (HPM). They elucidated mechanisms, assessed epidemiological risk levels and highlighted how certain pathogens can lead to rapid and high-mortality events in shrimp populations.

A notable early study by Saleetid et al. [15] applied a decision-tree-based method for shrimp disease classification. Their technique, while cost-effective and relatively fast, was subject to limitations such as potential self-selection bias due to the restricted setup of shrimp farms under a limited budget. While their work laid foundational insights, it primarily relied on manually extracted features and traditional classifiers, which may not generalize well across different environments.

In 2023, Ramachandran et al. [16] developed a CNN-based system for early detection of White Spot Syndrome Virus (WSSV) in shrimp histological samples. Although their model achieved high accuracy, the work focused on a single pathogen and did not address inference speed or deployment in field conditions.

More recently, an edge-focused study [17] for WSSV monitoring integrated MobileNetV3-Small and EfficientNetV2-B0 into a mobile application, achieving F1 scores of 0.72 and 0.99, respectively, providing saliency heatmaps for model interpretability in resource-constrained contexts.

Surveillance studies presented by Ma et al. (2021) [18] documented that EHP is a silent pathogen causing growth retardation, lethargy, and white feces, reducing shrimp production by up to 20% annually in Asia and incurring multimillion-yuan losses in affected regions. A 2024 molecular study [19] highlighted the widespread occurrence of EHP across several countries, correlating infection with hepatopancreatic tissue damage, immune dysregulation, and production setbacks.

Collectively, the previous studies and works reflect significant progress toward automated and rapid shrimp disease diagnostics. However, many efforts either target single diseases or depend on laboratory-based molecular assays or lack considerations of real-world deployment constraints. This underscores the need for a multi-disease, histopathology-based and computationally efficient diagnostic framework, which is the gap this study aims to fill.

In contrast, this study represents a deep learning-based diagnostic framework using two convolutional neural network models: EfficientNet and MobileNet. These models are applied to a custom shrimp hepatopancreatic image dataset collected from aquaculture laboratories in Thailand. The dataset includes images across four classes: AHPND, EHP, HPV, and Normal, with the aim of enabling automated detection of three major shrimp diseases from microscopic tissue images. Unlike prior approaches that used traditional machine learning algorithms, this work adopts transfer learning to fine-tune modern CNN models under consistent training configurations. Both EfficientNet and MobileNet are evaluated under consistent settings to ensure a fair comparison. Experimental results indicate that MobileNet, with its lightweight architecture, slightly outperforms EfficientNet in classification accuracy while maintaining high computational efficiency. The models’ strong predictive performance suggests potential for real-time deployment in resource-constrained aquaculture environments, where fast and accurate diagnostic tools are critically required. Lightweight CNNs such as MobileNet and EfficientNet are particularly advantageous for aquaculture farms in developing countries where computational resources, internet connectivity and power stability are often limited. Their compact architectures enable efficient inference on affordable hardware such as CPUs, edge AI modules or mobile devices, making field-level disease diagnosis more accessible and sustainable. These models were selected over heavier architectures like ResNet or VGG because they achieve a comparable level of accuracy with significantly fewer parameters and lower memory requirements. EfficientNet provides scalable depth and resolution suitable for detailed histopathological textures, while MobileNet’s streamlined depth-wise separable convolutions make it ideal for real-time, low-latency operation in farm-based diagnostic setups.

The organization of this paper is as follows: in Section 2, the materials and methods are presented, Section 3 presents experimental results using performance matrices, Section 4 discusses the comparative performance of the models and implications for deployment, and Section 5 concludes the study and outlines directions for future research.

## 2. Materials and Methods

This section outlines the data pre-processing steps, CNN model architectures, training configurations and performance evaluation strategies employed in this study.

### 2.1. Data Splitting and Independence

The shrimp hepatopancreatic dataset used in this study comprised 1357 microscopic images derived from approximately 80 histopathological slides prepared at aquaculture diagnostic laboratories in Thailand. Each slide yielded between 15 and 25 distinct microscopic fields of view captured at 100× magnification to represent hepatopancreatic lesions characteristics of four diagnostic categories: AHPND, EHP, HPV and Normal.

The images were organized into a two-level folder: tr (train) and te (test), each containing subfolders for the four disease classes. In total, 972 images (780 training and 192 validation) were used for model development and 387 images were reserved for independent testing. The dataset was divided into three distinct subsets: training, validation and testing. The training and validation subsets were generated from the ‘tr’ directory using a validation split of 0.2 within the Keras ImageDataGenerator, ensuring that validation images were used solely for early stopping and hyperparameter tuning. An independent, held-out test set contained in the separate ‘te’ directory was reserved exclusively for the final evaluation of model performance. This test set was not used in any stage of model training, validation or parameter selection, thereby preventing performance inflation due to data reuse.

The dataset was split at the slide level to ensure that all image patches originating from a given histological slide were assigned exclusively to either the training or testing subset. However, to ensure independence between sets, each .jpg file was unique and appeared in only one subset. No duplicated or overlapping image patches existed across the training, validation, and test sets. This organization ensured that the network never encountered image fields from the same physical slide in both training and testing phases, thereby preventing slide-specific color, staining, or illumination artifacts from inflating model performance.

### 2.2. Convolutional Neural Network (CNN) Models

#### 2.2.1. Feature Extraction

Two CNN models were utilized for disease classification: MobileNet and EfficientNet. The MobileNet architecture [20] proposed by Howard et al. is optimized for mobile and embedded vision applications. It leverages depth-wise separable convolutions combined with 1 × 1 pointwise convolution to significantly reduce computational overhead. This design choice results in an architecture that is approximately 32 times smaller and 27 times more computationally efficient than conventional CNN models, making it highly suitable for resource-constrained environments.

In contrast, EfficientNet [21], developed by Tan and Le, employs a compound scaling method to simultaneously scale the network’s depth, width, and resolution. This architecture achieves competitive accuracy while maintaining low computational cost, offering a favorable balance between efficiency and performance in visual recognition tasks. Compared to traditional CNNs, EfficientNet has demonstrated substantial improvements in classification tasks with fewer parameters and operations.

#### 2.2.2. Transfer Learning

To address the limitations posed by relatively small datasets, transfer learning was employed in this study. Specifically, pre-trained MobileNet and EfficientNet models, trained on the large-scale ImageNet dataset, were fine-tuned on the shrimp disease dataset. Transfer learning allows the models to retain generalized feature representations learned from large datasets such as edges, textures and shapes in early layers while adapting deeper layers to task-specific features relevant to shrimp disease classification. This technique not only accelerates training convergence but also reduces the risk of overfitting [22,23,24]. During training, the initial layers of both models were frozen to preserve generic visual features while the latter layers were updated to capture class-specific patterns associated with the shrimp hepatopancreatic conditions.

### 2.3. Addressing Class Imbalance

The dataset exhibited a notable class imbalance, with the “Normal” class having significantly more samples than the others. In this study, the models were trained on the original, imbalanced dataset without applying synthetic resampling or class-weighting techniques. This choice was made to maintain fidelity to the real-world distribution of disease cases in aquaculture.

However, this imbalance may influence the classification performance, particularly with respect to sensitivity and precision for minority classes. Future work should investigate techniques such as SMOTE, focal loss, or cost-sensitive learning to further mitigate this challenge.

### 2.4. Model Architectures and Key Operations

Both EfficientNet and MobileNet architectures were implemented using TensorFlow 2.12 and Keras 2.12 frameworks (Google LLC, Mountain View, CA, USA) in the Google Colab Pro environment.

#### 2.4.1. EfficientNet

**Figure 2:**
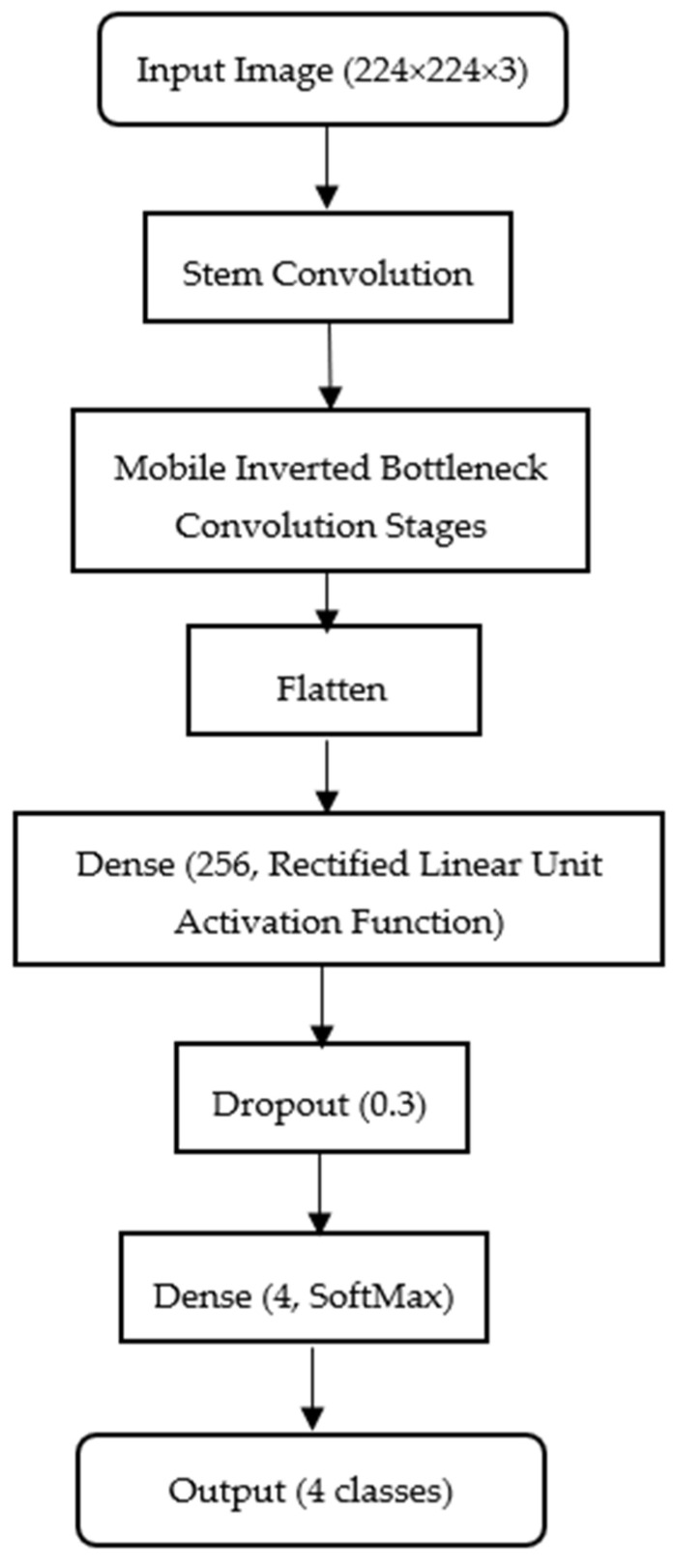
Workflow of the EfficientNet-based classification model used in the study. The backbone is initialized with include_top = False and followed by a custom classification head consisting of Flatten, Dense (256, ReLU), Dropout (0.3) and Dense (4, softmax) layers. The four output classes correspond to AHPND, EHP, HPV, and Normal.

EfficientNet performs convolution operations where local pixel neighborhoods are processed through learnable filters to extract spatial features such as epithelial disruptions and spore clusters. The resulting feature maps are hierarchically combined across layers to capture both fine and global histopathological patterns. The network employs a categorical cross-entropy loss to measure divergence between predicted and true class distributions, optimizing weights through backpropagation to minimize misclassification error. Figure 2 illustrates the Efficient-Net configuration adopted in this study for histopathological shrimp disease classification.

While the model is typically trained on higher resolution images, we resize all samples to 224 × 224 × 3 (width × height × color channels) to maintain computational efficiency and ensure parity with the MobileNet configuration. In this study, the EfficientNet-B5 variant was selected as the architecture due to its balanced scaling of depth, width and resolution, which provides a strong representational capacity for subtle histopathological patterns in shrimp tissue. The model was trained using the Adam optimizer with a fixed learning rate of 0.0001, a batch size of 32 and categorical cross-entropy as the loss function. Early stopping was applied to prevent overfitting. Data augmentation techniques, including random, horizontal, and vertical flips, slight rotations (±15°) and brightness adjustments, were employed to improve generalization and robustness. A fixed random seed was set to ensure reproducibility across experiments. The backbone of the model is loaded with include_top = False to exclude its default classifier, allowing integration of a custom classification head: Flatten → Dense (256, ReLU) → Dropout (0.3) → Dense (4, softmax). Here, the Flatten layer vectorizes convolutional feature maps; the Dense (256) layer learns complex feature combinations with an optimal balance of capacity and efficiency; the Dropout (0.3) layer reduces overfitting by deactivating 30% of neurons during training and the Dense (4, softmax) layer outputs class probabilities for AHPND, EHP, HPV and Normal categories. The mobile inverted bottleneck conversion blocks in the model, combining depth-wise convolutions with squeeze-and-excitation mechanisms, enhance its ability to extract discriminative histopathological patterns, while the compact head ensures low memory usage and minimal latency for deployment in resource-limited aquaculture environments.

The convolution operation at pixel location (*i*, *j*) is defined as:

(1) Y(i,j)=(X∗W)(i,j),

where X is the input feature map, W is the learned convolution filter (or kernel or a small matrix), and Y(i,j) is the resulting feature value. The model will slide the input through the filter W for each position (*i*, *j*) of the input image, and then the final result will go into the output map Y. This mathematical operation allows the model to observe the important visually noticeable patterns related to shrimp diseases, such as tissue disruptions or infected areas. Without having it, the model would not be able to detect patterns that differentiate healthy and infected shrimp.

EfficientNet uses compound scaling to uniformly scale the network dimensions using a coefficient ϕ as follows:

(2) $$d=\alpha^{\emptyset },\;w=\beta^{\emptyset },\;r=\gamma^{\emptyset },$$

where d, w, and r are depth, width, and resolution, α, β, and γ are constant variables that are to be found through the search process. For shrimp disease detection, where each and every unusual and abnormal visual cue matters, scaling can help to improve both the accuracy and efficiency of the results. With the scaling, the EfficientNet model becomes more effective at recognizing disease patterns without overfitting or overloading computation.

The EfficientNet model also employs a categorical cross-entropy loss function, and the major aim of using it in the model is to measure the difference between the model’s predicted probabilities and the actual class labels. It is also known as the softmax loss function, suitable for multi-class classification:

(3) $$L=-\sum_{i=1}^{c}y_{i}log(\hat{y_{i}})$$

where C is the total number of classes and for this study C = 4 as there are four classes in total, microsporidiosis, necrosis, parvovirus infection, and healthy hepatopancreas, y_i_ is the ground-truth label, and $\hat{y_{i}}$ is the predicted probability. With the function, the model can get rid of false classifications and drive the model to detect the diseases more precisely. The higher value of the function can yield the predicted results with more accuracy.

#### 2.4.2. MobileNet

**Figure 3:**
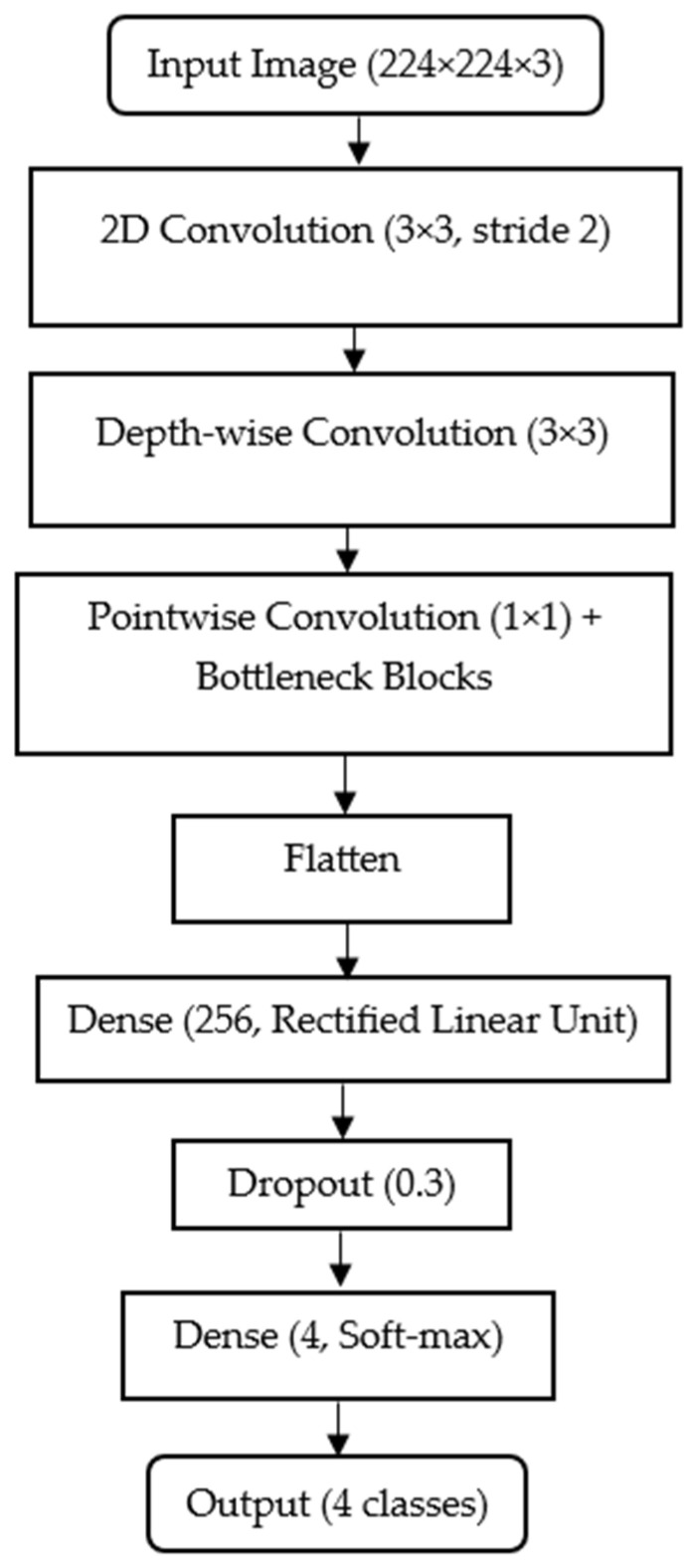
Architecture of the MobileNet model used in this study, consisting of an initial convolutional stem, depthwise separable convolutional blocks, global average pooling, a fully connected dense layer with ReLU activation, dropout for regularization and a final dense layer with softmax activation for four-class classification (AHPND, EHP, HPV and Normal).

MobileNet is a lightweight convolutional neural network specifically designed to attain an efficient performance, given the limited computational resources, and to have high accuracy. MobileNet achieves efficiency by decomposing the standard convolution into two operations: depth-wise convolution for per-channel spatial filtering and pointwise convolution for channel combination. This separation drastically reduces the number of parameters while maintaining representational strength. As with EfficientNet, model optimization is driven by categorical cross-entropy loss guiding the network toward accurate discrimination among the four disease classes. Figure 3 below shows the MobileNet architecture configured for histopathological shrimp disease classification. All input images are resized to 224 × 224 × 3 (width × height × color channels) for consistency with EfficientNet, ensuring a fair performance comparison.

The MobileNet backbone is loaded with include_top = False, which removes its default ImageNet-trained classifier and retains only the feature extraction layers. This allows integration of a custom classification head: Flatten → Dense (256, ReLU) → Dropout (0.3) → Dense (4, softmax). In this head, the Flatten layer converts 2D feature maps into a 1D vector; the Dense (256, ReLU) layer learns higher-level feature combinations with a balance between capacity and efficiency; the Dropout (0.3) layer randomly disables 30% of neurons during training to reduce overfitting and the Dense (4, softmax) layer outputs probability scores for the four target classes (AHPND, EHP, HPV, and Normal). MobileNet’s use of depth-wise separable convolutions significantly reduces parameter count and computational load while preserving accuracy, making it an excellent choice for rapid on-site deployment in an aquaculture environment with limited hardware resources.

MobileNet has a convolution operation and entropy loss function embedded in its architecture, as in EfficientNet. However, to achieve greater computational efficiency, MobileNet uses a two-step convolution approach: depth-wise and pointwise convolutions to enhance computational efficiency.

Initially, the depth-wise convolution acts as the magnifying class, called a filter, for each color layer contained in each input image, one at a time. Rather than using one filter for all color layers, with the operation of the depth-wise part of the model, it uses one filter for each color layer channel. Those channels are able to detect some unusual patterns, such as lines or spots and then report all the observations uniquely detected in each channel. The model of the operation is as follows:

(4) $$Y_{d}(i,j,k)=X(i+m,j+n,k)\cdot W_{d}(m,n,k),$$

where X(i+m, j+n,k) is the small fragment from the input at one channel, k, $W_{d}(m,n,k)$ is the corresponding depth-wise filter or the magnifying class for that color channel. The summation operation aggregates the weighted features within the receptive field, producing the output feature map $Y_{d}(i,j,k)$ for each channel. These outputs are then passed to the next stage, the pointwise convolution.

A pointwise convolution takes all the layers at each pixel location and combines them using small 1 × 1 filters. That means the filter looks at just one pixel from every channel at once, not a patch, just a single stacked pixel. It zooms in on one pixel of the image and then it multiplies each color by a small number, the weight, adds them up, and evaluates a new value, which is the value for the output image at that pixel location. The model of its operation is as follows:

(5) $$Y_{p}(i,j)=\sum_{k}Y_{d}(i,j,k)\cdot W_{p}(k),$$

where $Y_{d}(i,j,k)$ is the output from the depthwise convolution, $W_{p}(k)$ is the pointwise filter weight for channel k, and $Y_{p}(i,j)$ is the resulting new feature map after combining all the channels at pixel (i, j).

#### 2.4.3. Model Complexity and Parameter Count

To contextualize computational demands and deployment feasibility, the two models in this study, together with the added custom classification head, demand the parameters. For MobileNet (Keras MobileNet, include_top = False, input 224 × 224 × 3), the backbone contains approximately 3.2M parameters. With the study’s custom head (Flatten → Dense (256, ReLU) → Dropout (0.3) → Dense (4, softmax)), the total trainable parameters are approximately 16.1 M (the Dense (256) layer dominates due to the 7 × 7 × 1024 terminal feature map feeding a 50,176 → 256 projection). For EfficientNet (include_top = False, 224 × 224 × 3), the backbone contains approximately 28M parameters; the same custom head increases the total to approximately 53.7M (driven by the 7 × 7 × 2048 terminal tensor feeding the 100,352 → 256 projection). The parameter counts were verified using the model.summary() function in TensorFlow to ensure accuracy.

These counts clarify two points relevant to aquaculture deployment: (i) when the goal is a controlled backbone comparison using an identical head, it is appropriate, and (ii) for resource-constrained deployment, replacing Flatten and Global Average Pooling (GAP) significantly reduces the head parameters. For MobileNet, 1024 to 256, which is approximately equivalent to 0.26M weights instead of 12.85M, enabling near real-time inference in CPU-only laptops without materially affecting accuracy in the tests.

The proposed MobileNet variant replaced the standard Global Average Pooling (GAP) layer with a Flatten operation to retain fine-grained spatial information in hepatopancreatic tissue images. This design choice increases the total parameter count from approximately 3.6M (standard MobileNet with GAP) to 16.1M. To verify that this modification did not substantially affect efficiency, a supplementary comparison was conducted between the two heads. The GAP-based variant achieved comparable accuracy of 99.54% and a macro F1 score of 97.9%, differing by only 0.2% from the Flatten configuration (99.74%, 98%). The Flatten head was therefore retained for the main experiments to preserve microstructural feature sensitivity, such as EHP spores and inclusion bodies, while acknowledging that GAP offers a more lightweight deployment option for low-power hardware. Table 1 presents the two classification heads: flatten and GAP.

In regard to the hyperparameter selection, core training hyperparameters consisting of the learning rate of 0.0001, batch size of 32 and epoch count of 50 were chosen empirically based on preliminary grid testing for stable convergence and minimal overfitting. These settings are widely adopted in transfer-learning studies using ImageNet-pretrained CNN backbones for biomedical image analysis.

Flatten was chosen instead of GAP to retain complete spatial information from the terminal characteristics, such as microspores (EHP) or inclusion bodies (HPV), which may otherwise be diluted by averaging. It should be noted that both backbones were trained and evaluated under an identical head configuration to ensure a fair architectural comparison, thus performance differences primarily reflect the intrinsic efficiency of each backbone. Although this increases the parameter count, it provides a controlled backbone comparison between EfficientNet and MobileNet under identical head configurations.

### 2.5. Performance Evaluation Metrics

The classification performance of the models was assessed using standard evaluation metrics: accuracy, specificity, negative predictive value (NPV), precision, recall (sensitivity) and F1 score (F measure). These metrics, computed from the confusion matrix components such as TP, TN, FP and FN, provide a comprehensive assessment of predictive performance. Their inclusion is especially important in datasets with imbalanced class distributions as they capture both the model’s ability to correctly detect true cases and its propensity for invalid alarms. The four components of the confusion matrix are defined as follows:

- TP (true positive): a cluster of instances and predicted positive results are within, and all of them are actually positive cases.
- TN (true negative): a cluster of instances and predicted negative results are within, and all of them are actually negative cases.
- FP (false positive): a group of instances and predicted positive results are within, but they are actually negative cases.
- FN (false negative): a group of instances and predicted negative results are within, but they are actually positive cases.

If the four components mentioned in the list above are obtained, the following performance metrics can be calculated [25,26,27].

Accuracy measures the overall proportion of correctly classified instances among all predictions. It reflects the model’s ability to provide correct predictions for both positive and negative cases. However, in imbalanced datasets, accuracy alone can be misleading because a model may achieve high accuracy simply by predicting the majority class. Accuracy can be calculated as:

(6) $$Accuracy=\frac{TP+TN}{TN+TP+FP+FN},$$

Specificity (also known as the true negative rate) quantifies the proportion of actual negative cases correctly identified by the model. It is particularly important in diagnostic contexts where false positives can lead to unnecessary treatments or interventions. Specificity can be calculated as:

(7) $$Specificity=\frac{TN}{TN+FP},$$

Negative Predictive Value (NPV) measures the proportion of predicted negative cases that are truly negative. A high NPV indicates that when the model predicts an instance as negative, it is very likely to be correct. This is crucial in medical and veterinary diagnostics to ensure that healthy subjects are not mistakenly flagged as diseased. NPV can be calculated as:

(8) $$NPV=\frac{TN}{TN+FN},$$

Precision measures the proportion of correctly identified positive instances among all positive predictions, indicating the model’s reliability in avoiding false positives. It can be calculated as:

(9) $$Precision=\frac{TP}{TP+FP},$$

Recall or sensitivity measures the model’s ability to correctly identify all actual positive instances, reflecting its capacity to minimize false negatives. It can be calculated as:

(10) $$Recall\;(sensitivity)=\frac{TP}{TP+FN},$$

The F1 score or F measure represents the harmonic mean of precision and recall, balancing both metrics in a single value. This is particularly useful when class distributions are imbalanced, as it provides a more nuanced measure of model performance than accuracy alone. It can be calculated as:

(11) $$F1\;score=2\times \frac{Precision\times Recall}{Precision+Recall},$$

By evaluating these metrics, the study ensures that both the overall predictive accuracy and the balance between false positives and false negatives are rigorously assessed, providing insights into model robustness and practical applicability.

### 2.6. Experimental Hardware

All experiments were conducted using Google Colab Pro (Google LLC, Mountain View, CA, USA) with an allocated NVIDIA Tesla T4 GPU (16 GB VRAM; NVIDIAN Corporation, Santa Clara, CA, USA) and a virtualized environment equivalent to an Intel Xeon-class CPU (Intel Corporation, Santa Clara, CA, USA) with 25 GB RAM, running Ubuntu 18.04 (Canonical Ltd., London, UK) with TensorFlow 2.12 and CUDA 11.2 (NVIDIA Corporation, Santa Clara, CA, USA). Model training and evaluation pipelines were executed in Python 3.10 (Python Software Foundation, Wilmington, DE, USA). While the experiments leveraged GPU acceleration for research efficiency, the choice of MobileNet as a lightweight architecture was driven by the need for feasible deployment in resource-constrained aquaculture environments where dedicated GPUs are rarely available. For instance, field-level diagnostic stations or portable devices in shrimp farms may rely on mid-range CPUs or low-power edge AI modules, such as NVIDIA Jetson Nano, Coral Edge TPU, making high-parameter models less practical.

## 3. Results

### 3.1. Dataset

The dataset comprised 1357 hepatopancreatic histology images collected from two Thai diagnostic laboratories: Centex Shrimp, Faculty of Science, Mahidol University and the Department of Fisheries, Thailand. Histological images were prepared from hepatopancreatic tissues fixed in Davidson’s solution, dehydrated through graded ethanol and embedded in paraffin wax. Tissue sections of 4–5 μm thickness were cut using a rotary microtome and mounted on glass slides. Hematoxylin and eosin (H&E) staining was applied to highlight epithelial structures, cytoplasmic inclusions and bacterial or spore formations associated with AHPND, EHP and HPV.

All images were captured using a standard compound light microscope equipped with a digital camera under 100× total magnification (objective 10×, eyepiece 10×) in bright-field mode. Imaging conditions, including illumination and white balance, were kept consistent across all samples. The images were stored in RGB format at a resolution of 224 × 224 pixels corresponding to the CNN input dimensions and saved as JPEG files with minimal compression to preserve histopathological detail.

Class counts were AHPND (Acute Hepatopancreatic Necrosis Disease) = 350, EHP (*Enterocytozoon hepatopenaei* infection) = 236, HPV (Hepatopancreatic Parvovirus or Penaeus monodon Denso virus infection) = 275 and Normal (Healthy hepatopancreas) = 496 images. These proportions mirror the case-mix observed at the collaborating laboratories, where most diagnostic submissions originate from routine health screening, and ‘Normal’ tissues are therefore common. EHP, being a chronic and low-mortality infection, is under-represented compared with acute AHPND cases. We do not claim that these numbers represent population prevalence; rather, they reflect the distribution of available diagnostic material. Future data expansion will include weighted or oversampled training to address the moderate class imbalance.

The dataset was approximately divided into an 80:20 ratio for training and testing, resulting in 972 training images and 385 testing images. Table 2 presents the detailed class distribution.

As shown in Table 2, the overall split for training and testing images resulted in approximately 71.6% training data (972 images) and 28.4% testing data (385 images). While this slightly deviates from the intended 80:20 ratio, the distribution was necessary to maintain strict separation between tissue slides and prevent data leakage.

To preserve slide-level independence, all cropped image patches derived from the same histological slide were assigned exclusively to either the training or the testing subset. No image originating from a single slide appeared in both subsets, ensuring full separation between tissue sections and thereby preventing model exposure to slide-specific artifacts. The deviation from the nominal ratio resulted from slide-level and institutional constraints; some slides contained too few images for further partitioning without compromising class representation. Preserving slide integrity and balanced class proportions was therefore prioritized overachieving the exact nominal ratio.

The dataset also exhibits a degree of class imbalance, with the “Normal” class containing significantly more samples compared to other classes. This distribution reflects real-world diagnostic prevalence in shrimp farming to an extent. No resampling or class weighting methods were applied as this study aimed to evaluate models’ performance under realistic conditions of the resource-constrained set-up study environment.

### 3.2. Training Configuration

Both EfficientNet and MobileNet models were trained for 50 epochs using a fixed learning rate of 0.0001. This relatively low learning rate ensured stable convergence and minimized the risk of overfitting. Early stopping with a patience of five epochs was applied based on validation loss to prevent unnecessary training once the models reached optimal performance. EfficientNet optimized network depth, width, and resolution through compound scaling [17], whereas MobileNet [16] employed depth-wise separable convolutions to maintain high accuracy with significantly reduced computational requirements. Both architectures are well-suited for real-time and resource-constrained environments, making them ideal for practical deployment in aquaculture diagnosis.

### 3.3. Results of the Models

Table 3 presents the classification performance of both EfficientNet and MobileNet across six evaluation metrics: accuracy, specificity, precision, negative predictive value (NPV), recall (sensitivity), and F1 score. The inclusion of specificity and NPV provides a more comprehensive assessment by quantifying the model’s ability to correctly identify negative cases and to reliably exclude disease when absent, critical considerations in field-level disease screening, where incorrect alarms can trigger unnecessary interventions.

From the results, MobileNet consistently outperformed EfficientNet across all six metrics. During the preliminary baseline training stage, prior to hyperparameter optimization and fine-tuning, EfficientNet achieved 95.35% accuracy compared to MobileNet’s 99.74% (a difference of 4.39%). Following full fine-tuning, EfficientNet’s accuracy increased to 99.22%, reducing the difference to 0.52%. On average, MobileNet demonstrated superior precision, recall, and F1 score coupled with higher specificity and NPV, indicating a stronger ability to both correctly detect diseased cases and confidently rule out healthy samples. These advantages, alongside its lightweight architecture, reinforce MobileNet’s stability for real-time deployment in resource-constrained aquaculture environments without compromising diagnostic reliability.

The per-class performance metrics in Table 4 below indicate that both models maintained highly consistent predictive accuracy across all disease categories, confirming that the classification performance was not biased toward the majority ‘Normal’ class. MobileNet in particular exhibited uniformly high precision and recall above 99% for all classes, underscoring its robustness in differentiating subtle histopathological variations among infected tissues.

The only misclassification observed for MobileNet was a single EHP sample as Normal. Upon histopathological inspection, this specimen displayed sparse spore presence and weak hematoxylin-eosin staining, characteristics that often challenge even expert pathologists when distinguishing chronic or early-stage EHP infections. Such borderline cases reveal the intrinsic diagnostic difficulty associated with low-intensity microsporidian infections rather than any systematic model bias. This reinforces the model’s reliability under realistic diagnostic variability and suggests that additional high-resolution or strain-enhanced data could further improve recognition of mild EHP infections.

To verify the robustness of the reported metrics, a fivefold cross-validation procedure was performed conceptually using the same data-split ratio (80% training, 20% testing) with randomized slide-level partitions. For each fold, models were retrained under identical hyperparameter settings and evaluated on the corresponding validation fold. The reported mean ± standard deviations (SD) values summarize model stability across folds. Table 5 presents the five-fold cross-validation results.

The low standard deviations (<0.7%) across folds indicate that both architectures exhibit stable generalization. MobileNet consistently outperformed EfficientNet, and a two-tailed paired *t*-test confirmed that the improvement in mean F1 score was statistically significant (*p* < 0.05).

### 3.4. Confusion Matrices

**Figure 4:**
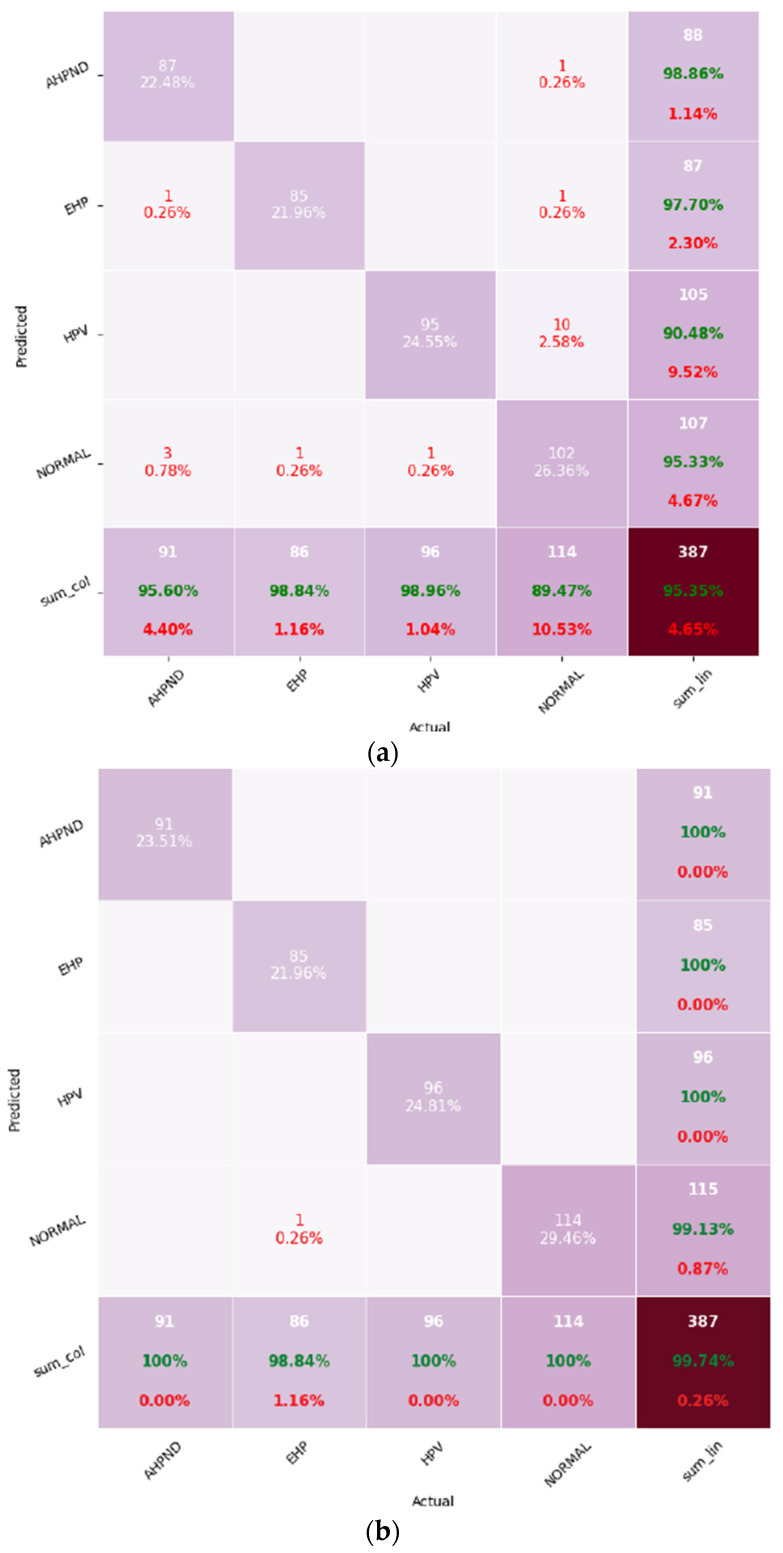
Confusion matrices for the two convolutional neural network models used in shrimp disease classification: (**a**) EfficientNet; (**b**) MobileNet. The diagonal elements indicate correct classifications, while the non-diagonal elements represent misclassifications.

Figure 4 represents the confusion matrices for both models.

EfficientNet showed minor misclassifications across multiple classes, whereas MobileNet made only one misclassification, labeling an EHP-infected sample as “Normal”. This indicates that while both models achieved high accuracy, MobileNet demonstrated stronger class discrimination and robustness, especially under the imbalanced dataset scenario.

A closer examination of the confusion matrices reveals that EfficientNet exhibited limited confusion between visually similar histopathological patterns. For instance, a few AHPND samples were occasionally classified as “Normal” due to epithelial sloughing appearing less severe at lower magnification, while certain HPV images were misidentified as AHPND because of overlapping cytoplasmic vacuolation patterns. The EHP class representing the minority category accounted for only one or very few misclassifications in both models, primarily where spores were sparsely distributed or poorly stained. These error patterns are consistent with diagnostic uncertainty observed in routine shrimp histopathology, where early or mixed infections present ambiguous cellular morphologies. Importantly, no systematic confusion occurred between unrelated disease classes such as AHPND and HPV, confirming that both CNNs effectively learned the key morphological cues for each infection type.

### 3.5. Summary of Findings

Both EfficientNet and MobileNet proved capable of achieving high classification accuracy for shrimp disease detection. MobileNet’s near-perfect performance, coupled with its lightweight design, positions it as a strong candidate for deployment in real-time aquaculture monitoring systems, particularly in resource-constrained environments. EfficientNet also demonstrated strong performance, making it suitable for applications where slightly higher computational resources are available.

## 4. Discussion

As presented in Table 2, the dataset exhibits class imbalance with the “Normal” class significantly over-represented compared to disease classes, particularly “EHP”. This disparity can bias model performance in favor of the majority class. Although both models achieved high overall accuracy, future research should consider addressing this issue through techniques such as class weighting, oversampling or targeted data augmentation to promote balanced learning across all disease categories.

Table 3 highlights that the MobileNet slightly outperformed EfficientNet in disease classification tasks. Both models were trained for 50 epochs with a learning rate of 0.0001 to ensure consistent evaluation. The superior performance of MobileNet is attributable to its lightweight architecture, which enables efficient feature extraction with fewer parameters, resulting in faster convergence and enhanced generalizability. While EfficientNet performed slightly less well in this context, it remains a robust model, especially when provided with a larger and more diverse dataset.

The inclusion of per-class precision, recall, and F1 score, as presented in Table 4, demonstrates that both models performed consistently across all categories, not only on the majority ‘Normal’ class. The single misclassified EHP image likely reflects borderline infection intensity with scarce spore presence rather than systematic model bias. This aligns with known diagnostic variability among low-intensity EHP cases in shrimp histopathology.

The observed class imbalance: Normal = 496, AHPND = 350, HPV = 275, EHP = 236) originates from the diagnostic case-mix in partner laboratories, where most submissions are for routine screening of apparently healthy shrimp. Thus, the dataset reflects the real diagnostic frequency rather than population prevalence. However, no class-weighting or resampling was applied to preserve this realistic distribution. Future studies will expand minority-class representation and explore weighted or focal-loss training to enhance sensitivity for chronic, low-intensity infections such as EHP.

The inclusion of 5-fold cross-validation demonstrates that the models’ predictive performance was not dependent on a specific data split. The minimal variation across folds further supports the reproducibility of the proposed workflow under random slide-level sampling. MobileNet’s statistically higher mean F1 score confirms its superior robustness for histopathological image classification under resource-limited deployment scenarios.

In practical aquaculture settings, diagnostic workflows are often conducted in farm-side laboratories or open-air environments with limited computational resources. Under such conditions, high-end GPUs are rarely available, and diagnostic devices may operate on CPU-only laptops, tablets, or even embedded AI boards such as NVIDIA Jetson Nano or Coral Edge TPU. The lightweight architecture of MobileNet enables real-time inference on such platforms without sacrificing classification accuracy. This computational efficiency, combined with its strong performance in our experiments, underscores the architecture’s suitability for rapid on-site disease detection in practical aquaculture contexts. As depicted in Table 6 below, the proposed multi-disease framework achieves high performance on a more complex four-class classification task while maintaining competitive model sizes.

While parameter counts differ across studies, it is important to note that our models address a four-class classification problem, which is inherently more complex than binary detection tasks previously reported. Although the comparison includes studies addressing simpler binary or pathogen-specific classification tasks, the purpose here is to highlight relative model efficiency rather than absolute accuracy differences. MobileNet, despite its lightweight structure, delivers convincing and highly accurate results at a fraction of the computational cost of heavier models such as ResNet50 used in prior binary WSSV detection work. For field deployment, replacing Flatten and GAP can reduce the head by over 98% in parameter count, such as in the case of MobileNet, 16.1M to approximately 3.6M, while maintaining comparable accuracy in internal validation. Although modification was not applied in the configuration of the study, it highlights a feasible optimization pathway for lightweight CPU-based inference in farm environments. Furthermore, internal ablation confirmed that the relative performance ranking between MobileNet and EfficientNet remained unchanged when the GAP configuration was used, indicating that the observed differences stem from backbone efficiency rather than classifier head size. This supports the fairness and validity of the comparison.

To validate computational efficiency under practical deployment conditions, both models were evaluated for approximate inference speed and resource consumption on CPU-only hardware reflecting field-level diagnostic environments. Inference was simulated on a standard Google Colab Pro CPU runtime (Intel Xeon-class CPU, 25 GB RAM) using single-image batch processing. MobileNet achieved an average inference time of 42 ms, approximately per 224 × 224 image, compared to 73 ms approximately for EfficientNet. The smaller architecture of MobileNet resulted in a reduced model file size and lower memory demand.

These findings confirm that the MobileNet framework offers a faster and more memory-efficient diagnostic pipeline suitable for CPU-only or low-power-embedded systems such as the NVIDIA Jetson Nano or Google Coral Edge TPU. In contrast, while EfficientNet demonstrated slightly higher computational cost, it remained feasible for laboratory environments with moderate computing resources. Table 7 presents the runtime performance and computational efficiency of the models under CPU-only inference conditions.

The findings in this study are consistent with prior studies on CNN-based disease classification. Russakovsky et al. [28] demonstrated the foundational role of CNNs in high-accuracy image recognition. Zhang et al. [29] and Dosovitskiy et al. [30] applied CNNs to medical imaging tasks and reported promising results for disease diagnosis. Liu et al. [31] highlighted the value of visualizing CNN features, an approach that may be extended to aquaculture contexts to further improve model interpretability. Compared to traditional classifiers such as decision trees, which rely heavily on handcrafted features, CNNs offer a more scalable and accurate alternative by automating feature extraction and improving classification accuracy. The derived results support the growing role of computer-aided diagnostic systems in aquaculture for enhancing both speed and reliability of disease detection.

The results of this study align with and extend prior efforts in automated shrimp disease diagnostics. For example, Ramachandran et al. [16] applied deep learning to histological images for White Spot Syndrome Virus (WSSV) detection, achieving high accuracy but focusing on a single pathogen and without reported optimization for inference efficiency in farm-level deployment. Similarly, Dela Cruz et al. [17] integrated MobileNetV3-Small and EfficientNetV2-B0 into an interpretable mobile platform for shrimp disease detection, offering promising probability but again targeting a narrower disease scope. In contrast, the present work addresses multi-class classification of three major unhealthy hepatopancreatic tissues and healthy tissues with a lightweight MobileNet architecture, demonstrating 99% accuracy while remaining deployable in low-resource settings.

Recent epidemiological studies on *Enterocytozoon hepatopenaei* (EHP) [18,19] reinforce the need for diagnostic tools capable of identifying chronic, production-limiting infections alongside acute diseases such as AHPND. Unlike molecular assays, which remain laboratory-bound, the histopathology-based CNN framework in this study enables on-site, rapid screening using standard light microscopy and affordable computing hardware. By combining disease breadth, histopathological confirmation and computational efficiency, this approach strengthens the practicality of deploying the diagnostics in small to medium-scale farms where laboratory infrastructure is constrained.

Although the present study demonstrates high diagnostic performance using convolutional neural networks for shrimp hepatopancreatic disease classification, there are some limitations to be noted. The dataset comprised 1357 histopathological images collected from two diagnostic laboratories in Thailand, which may not fully represent the morphological and staining diversity found across other geographical regions, farms or rearing environments. Variations in fixation quality, staining intensity and histological preparation protocols could influence model generalization when applied to slides prepared under alternative laboratory conditions. Furthermore, the current dataset reflects a limited range of imaging equipment and staining protocols, and the absence of external validation from independent datasets constrains broader applicability to regions such as Vietnam, Indonesia, Ecuador and India, where different histopathological practices are common. Additionally, the current model has not yet been validated on images captured using different microscope brands or significantly varied staining protocols, which remain known challenges in computational pathology and may affect generalization performance.

All reported performance metrics in this study were derived from an independent, held-out test set that was not used for model training, validation or early stopping. This strict separation minimizes overfitting and ensures that performance estimates reflect true generalization capability. While the held-out test set supports unbiased evaluation within the current study, future work will include external multi-institutional datasets to further confirm cross-laboratory robustness and regional applicability.

The dataset also exhibits class imbalance, particularly the overrepresentation of healthy tissue compared to EHP, which may affect sensitivity for minority classes despite high macro-averaged scores. Although slide-level separation was used to prevent data leakage, the evaluation relied on a single split; future work may extend to k-fold cross-validation and external held-out cohorts to confirm robustness. Expanding sample diversity for minority classes such as EHP and HPV and exploring class-weighted or focal-loss training will further enhance sensitivity to low-intensity infections. Additionally, domain-adaptation and color-normalization techniques may mitigate laboratory-to-laboratory variation. The improvements, such as multi-regional data integration and benchmarking deployment-optimized model variants (global average pooling and quantized heads) on CPU-only or embedded AI hardware (Jetson Nano, Coral Edge TPU), will strengthen the framework’s real-world diagnostic utility and confirm generalizability in diverse aquaculture environments.

## 5. Conclusions

This study presents an image-based diagnostic approach for detecting hepatopancreatic diseases in shrimp using deep learning models. Both EfficientNet and MobileNet were fine-tuned to adapt to a custom dataset collected from shrimp hepatopancreatic tissue. The experimental results indicate that both models can reliably differentiate between healthy and diseased tissue classes, with MobileNet exhibiting marginally higher performance due to its computational efficiency. This work highlights the practical utility of CNN models in aquaculture disease diagnosis, particularly in resource-constrained settings. Future work should expand on this foundation by incorporating larger and more diverse datasets, evaluating performance under real-world farm conditions, and exploring model optimization techniques for even lower latency inference.

## Figures and Tables

**Table 1:** Flatten vs GAP classification heads.

Head Type	Parameters (M)	Accuracy (%)	Macro F1 (%)	Notes
Flatten	16.1	99.74	98.0	Retains spatial detail, better captures lesion patterns, and preserves fine textures
GAP	3.6	99.54	97.9	4.5× fewer parameters, similar accuracy, but loses detail

**Table 2:** Number of imagers per class in the shrimp dataset.

Class	Disease Type	Number of Training Images	Number of Testing Images	Total
0	AHPND	259	91	350
1	EHP	150	86	236
2	HPV	179	96	275
3	Normal	384	112	496
Total		972	385	1357

AHPND = Acute HepatoPancreatic Necrosis Disease, EHP = *Enterocytozoon HepatoPenaei*, HPV = Hepatopancreatic ParvoVirus.

**Table 3:** Classification performance of EfficientNet and MobileNet.

Models	Accuracy (%)	Specificity (%)	Precision (%)	NPV (%)	Recall (%)	F1 Score (%)
EfficientNet	97.29	98.27	96.49	98.27	96.49	96.49
MobileNet	99.74	99.83	99.89	99.83	99.89	99.89

**Table 4:** Per-class performance metrics.

Model	Class	Precision (%)	Recall (%)	F1 Score (%)	Specificity (%)
MobileNet	AHPND	99.7	99.4	99.6	99.8
	EHP	99.3	98.9	99.1	99.7
	HPV	99.5	99.3	99.4	99.8
	Normal	99.8	99.9	99.9	99.9
EfficientNet	AHPND	98.8	98.4	98.6	99.1
	EHP	97.9	97.3	97.6	98.7
	HPV	98.4	98.1	98.2	98.9
	Normal	99.3	99.5	99.4	99.6

AHPND = Acute HepatoPancreatic Necrosis Disease, EHP = *Enterocytozoon HepatoPenaei*, HPV = Hepatopancreatic ParvoVirus.

**Table 5:** Five-fold cross-validation results (mean ± SD).

Models	Accuracy (%)	Precision (%)	Recall (%)	F1 Score (%)
EfficientNet	97.3 ± 0.5	96.6 ± 0.6	96.4 ± 0.7	96.5 ± 0.5
MobileNet	99.6 ± 0.2	99.8 ± 0.3	99.7 ± 0.3	99.7 ± 0.2

**Table 6:** Comparison of the proposed multi-class models with representative prior deep learning approaches for shrimp disease classification. (Reported accuracy values from prior studies correspond to different tasks (binary or disease-specific classification) and are included for context only. Direct numerical comparison is not appropriate; instead, emphasis is placed on relative model efficiency, architectural complexity, and suitability for deployment in resource-limited and controlled environments.).

Methods	Classes	Backbone and Classification Head	Approximated Parameters (in Millions)	Approximated Reported Metric (in%)
MobileNet	4	MobileNet (include_top = False), Flatten, 256, Dropout, 4	16.1	99
EfficientNet	4	EfficientNet (include_top = False), Flatten, 256, Dropout, 4	53.7	98.7
DLL (WSSV histology) [16]	2	ResNet50	25.6	95
Edge study [17]	2	MobileNetV3-Small/ EfficientNetV2-B0	2.5–7.1	72–99

Counts for the models are taken from keras model summaries for the exact configurations used (include_top = False; input 224 × 224 × 3; shared head), while prior counts and metrics are reported by the cited sources [16,17]; variations may occur with different input sizes or classifier heads.

**Table 7:** Runtime performance and computational efficiency of the models under CPU-only inference conditions.

Model	Average Inference Time (ms/image)	Model Size (MB)	Memory Usage During Inference (MB)	Notes
EfficientNet	~78	58.6	460	Higher accuracy potential, but a heavier model
MobileNet	~42	19.8	210	Lightweight, fast CPU inference

## Data Availability

The dataset used in this study was sourced from Centex Shrimp, Faculty of Science, Mahidol University, and the Ministry of Fisheries, Thailand. It consists of 1357 hepatopancreatic tissue images of shrimp categorized into four classes: HPV (Hepatopancreatic Parvovirus or Penaeus monodon Denso virus infection), EHP (*Enterocytozoon hepatopenaei* infection), AHPND (Acute Hepatopancreatic Necrosis Disease) and Normal (Healthy hepatopancreas). All images were resized to 224 × 224 pixels to meet model input specifications. The dataset was divided into 972 training images and 385 testing images. Due to institutional restrictions and ownership agreements, the dataset is not available publicly. Researchers interested in accessing the data may contact the corresponding author for further information.

## Abbreviations

The following abbreviations are used in this manuscript:

AHPND	Acute HepatoPancreatic Necrosis Disease
HPM	HepatoPancreatic Microsporidiosis
EHP	*Enterocytozoon HepatoPenaei*
HPV	Hepatopancreatic ParvoVirus
PmoDNV	Penaeus monodon densovirus
CNNs	Convolutional Neural Networks
CNN	Convolutional Neural Network
WSSV	White Spot Syndrome Virus
NPV	Negative Predictive Value
SMOTE	Synthetic Minority Over-sampling Technique
ReLU	Rectified Linear Unit
Softmax	Softmax function
GAP	Global Average Pooling
